# TrIPP: a trajectory iterative p*K_a_* predictor

**DOI:** 10.1093/bioinformatics/btag063

**Published:** 2026-02-12

**Authors:** Christos Matsingos, Ka Fu Man, Arianna Fornili

**Affiliations:** Department of Chemistry, School of Physical and Chemical Sciences, Queen Mary University of London, Mile End Road, London E1 4NS, United Kingdom; Saints-Pères Paris Institute for the Neurosciences, Université Paris Cité, Paris 75270, France; Department of Chemistry, School of Physical and Chemical Sciences, Queen Mary University of London, Mile End Road, London E1 4NS, United Kingdom; Department of Chemistry, School of Physical and Chemical Sciences, Queen Mary University of London, Mile End Road, London E1 4NS, United Kingdom

## Abstract

**Summary:**

The protonation propensity of ionizable residues in proteins can change in response to changes in the local residue environment. The link between protein dynamics and p*K*_a_ is particularly important in pH regulation of protein structure and function. Here, we introduce TrIPP (Trajectory Iterative p*K*_a_ Predictor), a Python tool to track and analyze changes in the p*K*_a_ of ionizable residues along Molecular Dynamics trajectories of proteins. We show how TrIPP can be used to identify residues with physiologically relevant variations in their predicted p*K*_a_ values during the simulations and link them to changes in the local and global environment.

**Availability and implementation:**

TrIPP is available at https://github.com/fornililab/TrIPP.

## 1 Introduction

The conformation of proteins can depend on different factors, including the pH. Indeed, several examples exist of proteins that undergo structural shifts in response to physiological pH changes (Di Russo *et al.* 2012, Schönichen *et al.* 2013, Warwicker 2022, Matsingos *et al.* 2024). The link between pH and protein structure is mediated by the dependence between p*K*_a_ values, which regulate the protonation propensity of ionizable residues, and the local residue environment, which can significantly change with the protein conformation.

Due to the difficulty in experimentally measuring p*K*_a_ values, different computational methods (Rabenstein and Knapp 2001, Gordon *et al.* 2005, Alexov *et al.* 2011, Olsson *et al.* 2011, Anandakrishnan *et al.* 2012, Reis *et al.* 2020, Gokcan and Isayev 2022, Wei *et al.* 2023) have been developed to predict p*K*_a_ deviations from their reference values (isolated amino acid in solution). These approaches are normally applied to single structures, e.g. to define the protonation state of ionizable residues before starting a Molecular Dynamics (MD) simulation. Few studies have also considered p*K*_a_ calculations over structural ensembles to take into account that structural variations observed during MD simulations can influence predicted p*K*_a_ values (Lee *et al.* 2014, Matsingos *et al.* 2024). Building on this multiple-structure perspective, we introduce TrIPP (Trajectory Iterative p*K*_a_ Predictor), a tool to track, analyze, and visualize p*K*_a_ changes when postprocessing MD trajectories.

TrIPP is based on the iterative use of PROPKA 3 (Olsson *et al.* 2011), one of the most popular empirical approaches for single-structure p*K*_a_ prediction. In addition, TrIPP provides optional support to run the deep learning predictor pKAI (Reis *et al.* 2022), trained on Poisson Boltzmann-based predictions from PypKa (Reis *et al.* 2020). We show that TrIPP can be used to visualize p*K*_a_ distributions and time evolutions over MD trajectories and identify residues with conformation-dependent p*K*_a_ values. Pseudo-mutations can be carried out to assess the influence of specific residues on p*K*_a_ regulation. In addition, TrIPP can cluster trajectory frames according to the local environment of selected ionizable residues, to identify structures representative of the environments leading to different p*K*_a_ values. Insights from TrIPP can be used to guide further studies and formulate mechanistic hypotheses on pH-dependent regulation of protein function.

## 2 Implementation

TrIPP has been implemented using Python 3.9 along with several Python packages, including PROPKA 3.5.0 (Olsson *et al.* 2011), pKAI 1.2.0 (Reis *et al.* 2022), MDAnalysis 2.5.0 (Michaud‐Agrawal *et al.* 2011, Gowers *et al.* 2016), Pandas 2.0.3 (McKinney 2010), NumPy 1.25.0 (Harris *et al.* 2020), scikit-learn 1.5.0 (Pedregosa *et al.* 2011), scikit-learn-extra 0.3.0, scipy 1.13.0 (Virtanen *et al.* 2020), and seaborn 0.13.2 (Waskom 2021). The software has been packaged with Poetry 1.7.1 and is available for installation through PyPI.

### 2.1 TrIPP workflow

**Figure 1:**
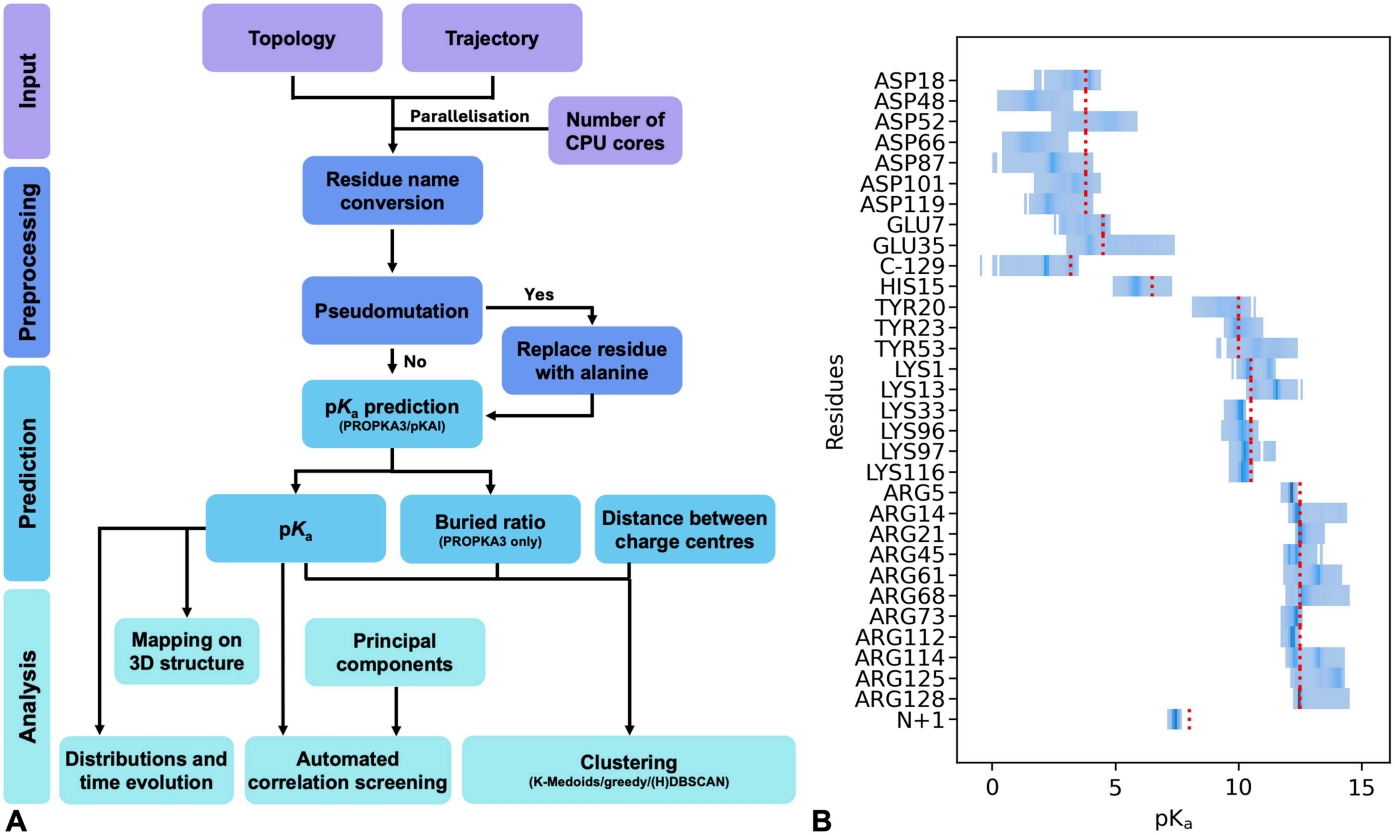
TrIPP overview and application to the hen egg white lysozyme. (A) Different stages in the TrIPP workflow. (B) Distributions of predicted PROPKA p*K*_a_ values for ionizable residues in lysozyme during one of the trajectories (MD1). For each residue, p*K*_a_ values are binned along the *x*-axis and more frequent values are indicated with a darker shading. A dotted vertical line indicates the model value for the corresponding residue type.

The workflow comprises four stages: data input, data pre-processing, p*K*_a_ prediction, and data analysis (Fig. 1A).

*Data input.* TrIPP uses MDAnalysis to load input trajectory and topology files. Parallelization is carried out by splitting each trajectory into as many subsets of frames as the number of cores used in the calculation. Each subset is processed by a different core in the subsequent steps.

*Data preprocessing.* Preprocessing includes an optional pseudo-mutation feature, which can be activated by the user to replace selected residues with alanine. Where required, residue names are also converted into versions recognized by PROPKA. Each snapshot is then temporarily converted to a PDB file, which is the format required by PROPKA.

*Prediction.* The chosen p*K*_a_ predictor (PROPKA by default) is iteratively run on each snapshot (via Python API) and its output is parsed to extract the p*K*_a_ values and (optionally only for PROPKA predictions) the buried ratio (given as %) of all the ionizable residues. Temporary files (e.g. PDB snapshots and PROPKA output) are deleted after processing. The extracted values are stored in CSV files and sorted by snapshot timeframe.

*Data analysis.* Plots of p*K*_a_ distributions and time evolutions can be generated from the p*K*_a_ values predicted for each snapshot. TrIPP can write PyMOL sessions (PSE format) to colour-map different properties on the 3D structure, including the p*K*_a_ values of all the ionizable residues for a selected frame or averaged over a set of trajectories. The difference between the average and reference model p*K*_a_ values used by the specific predictor can be also mapped. If the projection of the trajectories on selected principal components is provided by the user, an automatic scanning of the correlation coefficients between projections and p*K*_a_ values can be run to identify possible p*K*_a_-collective motions coupling.

The generated data can be used to extract representative structures from the trajectory using one of the available clustering algorithms: K-Medoids (Kaufman 2005), greedy (Micheletti *et al.* 2000), DBSCAN (Ester *et al.* 1996), and HDBSCAN (Campello *et al.* 2013). The feature matrix used for the clustering is built from p*K*_a_ values of user-selected residues and (optionally) their buried ratio (PROPKA only) and inter-residue distances. Features are Z-score normalized before clustering and (optionally) subjected to Principal Component Analysis (PCA). For each method, clustering hyperparameters can be optionally optimized via grid search, using silhouette widths (Rousseeuw 1987) to assess the quality of the clustering.

At last, TrIPP provides a function to automatically scan all ionizable residues for possible correlations between their p*K*_a_ values and a time-dependent property provided by the user. For example, projections from a principal component analysis of the trajectories can be used to represent collective motions in the protein.

### 2.2 TrIPP classes

TrIPP provides its functionalities through 3 main classes: *Trajectory* (input, preprocessing and prediction), *Visualization* (3D mapping of p*K*_a_- related values) and *Clustering*. A comprehensive tutorial demonstrating the use of these classes is included in the TrIPP github distribution as Jupyter notebook.

## 3 Application

TrIPP was applied to MD simulations of the hen egg-white lysozyme to illustrate its features. This enzyme was chosen since the p*K*_a_ values of its residues have been extensively studied both experimentally and computationally (Williams *et al.* 2010, Webb *et al.* 2011). In particular, the p*K*_a_ of acidic residues in its active site has been shown to be modulated by interactions with nearby residues (Williams *et al.* 2010). MD simulations were run for 300 ns (production) in five replicates (MD1–MD5), system preparation and MD protocol are described in the Supplementary Methods and Table 1, available as supplementary data at *Bioinformatics* online. TrIPP was run on each replica using frames sampled every 100 ps and PROPKA as predictor.

**Figure 2:**
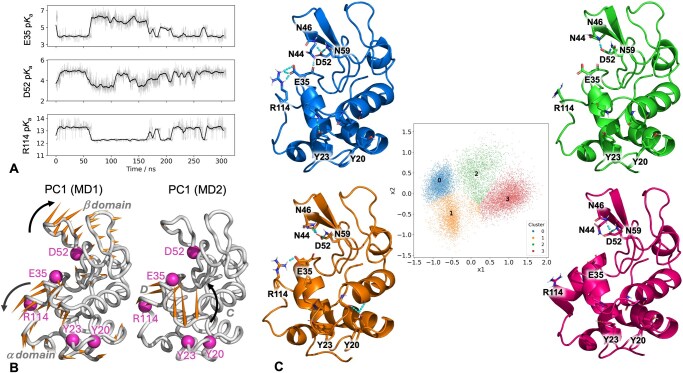
TrIPP-based analysis of MD simulations of lysozyme. (A) Time evolution of p*K*_a_ values predicted for Glu35, Asp52, and Arg114 during one of the lysozyme trajectories (MD1). The running average (5-ns window, dark shade) is shown together with the instantaneous values (light shade). (B) Porcupine representation of the first Principal Component (PC1) from the MD1 and MD2 lysozyme trajectories. Orange spikes show the direction and relative amplitude of motion of each residue along the PC (spikes shorter than 20% of the maximum length are omitted). Key residues are labelled and their position indicated with magenta spheres. Additional labels indicate the α and β domains in the left panel, and the C and D helices in the right panel. (C) K-Medoid cluster representatives (cartoon) illustrating different structural environments leading to different p*K*_a_ values for Glu35, Asp52, Arg114, Tyr20, and Tyr23. Key residues are highlighted as sticks and labelled. Polar contacts from PyMOL are represented with dashed lines. The feature matrix used for clustering was first subjected to dimensionality reduction via a Principal Component Analysis. The middle panel shows a projection of all trajectories on the first two principal components *x*_1_ and *x*_2_, with each frame coloured according to its cluster ID.

Average p*K*_a_ values show small variations across replicas, indicating good reproducibility, and a good agreement with the available experimental values (Table 2, available as supplementary data at *Bioinformatics* online). Inspecting the distribution of p*K*_a_ values observed for all ionizable residues (Fig. 1B and Fig. 1, available as supplementary data at *Bioinformatics* online) highlights the residues that have p*K*_a_ values (blue shades) significantly shifted from their model p*K*_a_ (dotted line). Among the acidic residues, Asp52 and Glu35 stand out because their p*K*_a_ values are upshifted towards the region of physiological pH. This can be also easily spotted in the PyMOL visualizations of the average p*K*_a_ values for acidic residues and their deviation from model values (Fig. 2, available as supplementary data at *Bioinformatics* online), where Asp52 and Glu35 are the only acidic residues with an increase in the average p*K*_a_ from the model values. While the average increase is small, inspection of the p*K*_a_ time evolution for these residues indicates that values can be as high as ∼7 for Glu35 and almost 6 for Asp52 (Fig. 2A). Interestingly, Glu35 and Asp52 are located in the lysozyme active site and are directly involved in the catalytic mechanism (Ramos *et al.* 2025).

It is worth noting that these simulations were performed using conventional MD, in which the protonation states of ionizable residues are fixed. Comparing the p*K*_a_ values sampled during the simulations with the range over which both protonation states are expected to have significant populations (grey shaded region in Fig. 1, available as supplementary data at *Bioinformatics* online) can flag residues for which alternative fixed protonation states or constant-pH MD (Oliveira *et al.* 2022) may be warranted, to ensure that conformational sampling remains consistent with protonation propensities over the course of the simulation. Notably, a subset of frames falls within this range for Glu35 (Fig. 1, available as supplementary data at *Bioinformatics* online), indicating appreciable population would be expected for the protonated form, consistent with protonation of this residue during catalysis (Ramos *et al.* 2025).

To detect possible couplings between p*K*_a_ changes and protein dynamics, the correlation coefficient between the p*K*_a_ time evolution and the main collective motions observed during the simulations (as described by projections of each trajectory on its top-ranking principal components) was calculated for each ionizable residue (Fig. 3, available as supplementary data at *Bioinformatics* online). The largest correlations (0.5 or higher) with the first two principal components (PC1 and PC2) were observed for Tyr20, Glu35, Asp52, Arg114, and to a less extent Tyr23, suggesting that the dominant protein motions can modulate the p*K*_a_ value of these residues.

Two different types of recurring motions were identified. The first one, exemplified by PC1 from MD1 (Fig. 2B, left), is the previously observed hinge-bending motion of the α- and β-domains(Brooks and Karplus 1985), where Asp52, Glu35, Arg114 are part of or close to the moving regions. The projections of the different trajectories (Fig. 4A, available as supplementary data at *Bioinformatics* online) show patterns that are clearly correlated (Glu35) or anti-correlated (Asp52, Arg114) with p*K*_a_ time evolutions (Fig. 5, available as supplementary data at *Bioinformatics* online). PC1 from MD2 instead describes a more localized restructuring of the loop between helices C and D, which is positioned above Tyr20 and Tyr23 (Fig. 2B, right).

Trajectories were then clustered to identify structures representative of the different environments around residues with motion-sensitive p*K*_a_ values (Fig. 2C). Clusters 0 and 1 feature a salt-bridge between Glu35 and Arg114, which is instead absent in clusters 2 and 3. This explains their distinct p*K*_a_ distributions across the clusters (Fig. 6, available as supplementary data at *Bioinformatics* online), since acidic and basic residues forming salt bridges are expected to have down- and up-shifted p*K*_a_ values, respectively, compared to their non-interacting states. Clusters 1 differs from cluster 0 because of the presence of additional hydrogen bonding between Asp52 and Asn44, which explains the lower Asp52 p*K*_a_ values for this cluster, and between Tyr20 and the CD loop backbone, which leads to increased Tyr20 p*K*_a_ values.

The environment of Glu35 and Asp52 was further analyzed using the pseudo-mutation tool implemented in TrIPP, where p*K*_a_ values are re-calculated after replacing selected residues with alanine, while keeping all the other coordinates in the protein unchanged. It is important to note that the pseudo-mutation feature is meant to help tracking the contribution of nearby residues to p*K*_a_ shifts from model values in the original (wild type) protein, rather than providing estimates of p*K*_a_ in mutants. Such quantitative assessment would require directly sampling the mutant ensemble with new MD simulations.

Replacing residues Asn44, Asn46 and Asn59 with alanine shows an increase of Asp52 p*K*_a_ across all the replicas (Fig. 7A and C, available as supplementary data at *Bioinformatics* online). This is consistent with the formation of hydrogen bonds between all these residues and Asp52. Interestingly, instead of just shifting the Asp52 p*K*_a_ time evolution profile, Asn44 also modulates its shape. Indeed, the Asn44Ala pseudo-mutant shows smaller p*K*_a_ fluctuations compared to the wild type (Fig. 7C, available as supplementary data at *Bioinformatics* online), consistently with the formation and breaking of Asn44-Asp52 hydrogen bonds during the trajectory. The role of Asn44 in stabilizing Asp52 negative charge and modulating its availability for substrate binding has been highlighted in recent structural studies of lysozyme (Ramos *et al.* 2025). Similarly, replacing Arg114 with alanine induces an increase of the Asp52 p*K*_a_ but only in the parts of the trajectory where the two residues are interacting with each other (Fig. 7B and D, available as supplementary data at *Bioinformatics* online). These examples illustrate how analyzing the effect of pseudo-mutations on the shape of the p*K*_a_ time evolution profile of a given residue can help identifying the residues that mediate the dynamic response of its p*K*_a_ value.

## 4 Concluding remarks

We developed a Python tool to track, analyze and visualize changes in p*K*_a_ values of ionizable residues in the post-processing of MD trajectories.

Information from TrIPP can be used to guide protein modeling, especially when a pH sensing behaviour is known or suspected (Matsingos *et al.* 2024). It is important to highlight that TrIPP is not meant to replace in any way constant-pH simulations (Williams *et al.* 2010, Oliveira *et al.* 2022), where the protonation state of ionizable residues is allowed to change. Here, we have shown how TrIPP can be used in conjunction with conventional MD simulations. While the sampled frames will reflect fixed protonation states, detection of p*K*_a_ changes during the trajectory can highlight residues that might require further simulations with alternative protonation states. Observing large correlations between residues p*K*_a_ and global motions might suggest that pH modulation of protein conformation is involved, warranting further investigation. Moreover, clustering and pseudo-mutation analyses can be used to build testable hypotheses about the molecular mechanisms underlying pH-dependent structural and functional changes.

**Note on related software.** An open-source Python implementation for running PROPKA across MD trajectory frames is also available (Irfan Dotson *et al.* 2020). Complementary features of TrIPP are support for parallel execution and for an alternative p*K*_a_ prediction engine (pKAI), residue-name preprocessing that allows for ligand containing systems and an extensive downstream analysis framework including pseudo-mutations and clustering of p*K*_a_-related features as described in this contribution.

## Supplementary Material

btag063_Supplementary_Data

## Data Availability

TrIPP source code and tutorial files are available at https://github.com/fornililab/TrIPP.
